# Berberine targets the STAT3 signaling pathway to improve cognitive impairment in chronic cerebral hypoperfusion rats

**DOI:** 10.3934/Neuroscience.2026005

**Published:** 2026-02-27

**Authors:** Chang Liu, Ying Gao

**Affiliations:** Department of Rheumatology and Immunology, The Third Affiliated Hospital of Jinzhou Medical University, No.2, Section 5, Heping Road, Linghe District, Jinzhou 121000, Liaoning Province, China

**Keywords:** chronic cerebral hypoperfusion, chronic cerebral ischemia, berberine, JAK2/STAT3, hippocampus, apoptosis

## Abstract

Berberine (BBR) possesses varied pharmacological properties, including anti-apoptotic and potent neuroprotective effects, and can ameliorate cognitive impairments associated with diverse diseases. Despite the noted potential of BBR in mitigating cognitive deficits associated with chronic cerebral hypoperfusion (CCH), the precise mechanisms underlying its therapeutic effects remain inadequately defined. To explore these mechanisms, a CCH rat model was developed using a refined micro-spring method for bilateral common carotid artery stenosis (BCAS). For the experimental setup, rats were systematically divided into six groups: a Sham group (n = 15), a Sham + BBR group (n = 15), a BCAS group (n = 15), a BCAS + BBR group (n = 15), a BCAS + BBR + Colivelin group (with Colivelin serving as a STAT3 activator, n = 15), and a BCAS + AG490 group (AG490 acting as a JAK2 inhibitor, n = 15). Cognitive performance was evaluated through the Morris water maze and novel object recognition (NOR) tests. Additionally, neuronal integrity was assessed by Nissl and TUNEL staining within the hippocampal region. The study further examined the protein expressions of JAK2, STAT3, phosphorylated JAK2, phosphorylated STAT3, and cleaved caspase-3 using western blot analysis. Interaction targets of BBR were predicted through the STITCH database, and its binding affinity to STAT3 was confirmed using molecular docking and surface plasmon resonance (SPR) techniques. The findings indicated an increase in apoptosis and a decline in cognitive abilities among the hippocampal neurons of the BCAS model rats. These deleterious effects, however, were substantially alleviated following treatment with BBR. The study posits that BBR primarily exerts its neuroprotective effects through the inhibition of the JAK2/STAT3 pathway. Notably, while the activation of this pathway by Colivelin exacerbated neuronal damage and cognitive decline, its inhibition via AG490 markedly decreased apoptosis and improved cognitive outcomes. Therefore, this research suggests that BBR enhances cognitive functions in BCAS rats predominantly by reducing apoptosis in hippocampal neurons through the modulation of the JAK2/STAT3 pathway.

## 1. Introduction

Chronic cerebral hypoperfusion (CCH), also known as chronic cerebral ischemia, is a widespread form of cerebral ischemic injury that plays a pivotal role in the onset of various neurological and cognitive disorders, such as Alzheimer's disease (AD) [1],[2]. Research has demonstrated that prolonged inadequate cerebral perfusion results in disruptions in energy metabolism, oxidative stress, release of inflammatory factors, neuronal apoptosis, and cognitive impairment [3]–[5]. With advancements in traditional Chinese medicine, traditional Chinese medicine extracts have shown significant efficacy in treating CCH [6]–[8].

Berberine (BBR), an isoquinoline alkaloid found in numerous plants such as Berberidaceae, has the molecular formula C_20_H_18_NO_4_. Initially extracted for its heat-clearing, detoxifying, and antibacterial properties, it is commonly used in clinical settings [9]. Extensive research has unveiled the wide-ranging pharmacological properties of BBR, including its antibacterial, anti-aging, and robust neuroprotective effects, positioning it as an ideal candidate for the treatment of cardiovascular and cerebrovascular diseases, diabetes, and multiple chronic inflammatory disorders. These findings have sparked considerable interest in the cardiovascular, cerebrovascular, and neurology research communities [10]–[13]. Current investigations into BBR have predominantly addressed acute cerebrovascular incidents and neurodegenerative diseases, with a focus primarily on AD [14],[15]. Conversely, research on CCH remains relatively scant. Prior studies have established that BBR can permeate the blood–brain barrier, which provides a substantial pharmacological foundation for its application in BCAS models [16],[17]. Evidence supports that BBR significantly improves learning and memory capabilities in AD models by attenuating apoptosis in hippocampal neurons [18],[19].

This study aimed to develop a BCAS rat model to investigate the therapeutic effects and underlying mechanisms of BBR on hippocampal neuron apoptosis. Behavioral tests, TUNEL staining, and western blotting were conducted to assess these effects, and the molecular interactions of BBR in the JAK2/STAT3 pathway were explored. The goal was to elucidate the pathways through which BBR mitigated cognitive impairment in BCAS models, thereby providing a foundation for identifying potential pharmacological targets for the treatment of CCH.

## 2. Materials and methods

### 2.1. Experimental animals

A total of 120 male Wistar rats, ranging in weight from 200 to 260 g and 8–10 weeks old (purchased from Beijing Weitonglihua Experimental Animal Technology Co., Ltd.), were housed in a 12-h light/dark cycle environment with free access to food and water, at a temperature of 22 ± 2 °C and humidity of 60% ± 5%. Rats were selected for this study due to their larger blood vessels and better-developed collateral circulation compared to mice, which are essential for accurately modeling chronic cerebral hypoperfusion (CCH). While rats exhibited a more robust collateral circulation than mice, the BCAS model has been validated for inducing chronic hypoperfusion in rats, with resultant neurological deficits well-documented in the literature.

### 2.2. Establishment and grouping of the BCAS model

Rats were randomly assigned to six experimental groups, as follows: Sham group (n = 15) [rats undergo the same surgical procedures but without BCAS (sham surgery)]; Sham + BBR group (n = 15) [sham rats treated with BBR (30 mg/kg/day) via intraperitoneal injection]; BCAS group (n = 15) (rats subjected to BCAS surgery without any treatment); BCAS + BBR group (n = 15) [BCAS rats treated with BBR (30 mg/kg/day) via intraperitoneal injection]; BCAS + BBR + Colivelin group (n = 15) [BCAS rats treated with BBR (30 mg/kg/day) via intraperitoneal injection and Colivelin (a STAT3 activator) administered intracerebroventricularly (2 µL of 10 mmol/L solution)]; and BCAS + AG490 group (n = 15) [BCAS rats treated with AG490 (a JAK2 inhibitor) administered intracerebroventricularly (2 µL of 10 mmol/L solution)].

The CCH rat model was created using the BCAS method [20]. Prior to surgery, rats were fasted for 12 h and anesthetized with an intraperitoneal injection of 3% pentobarbital sodium. Rats were placed on an experimental platform, and their subcutaneous tissues were separated. Bilateral common carotid arteries were dissected and constricted using a microcoil to create a CCH animal model. After surgery, wounds were sutured and disinfected, and penicillin was administered intramuscularly to reduce inflammation. Post-surgery, rats exhibited motor disorders and neurological weakness consistent with the model. The microcoils used for the BCAS surgery were manufactured by Sawane Spring Co., Ltd. (Japan), with an inner diameter of 0.2 mm, an outer diameter of 0.5 mm, and a length of 3 mm. These microcoils were specifically designed for use in small animal surgeries, allowing for precise and consistent constriction of blood vessels. The small dimensions and flexible material of the microcoils ensure that they can be tightly wrapped around the bilateral common carotid arteries without causing excessive damage to surrounding tissues. This allows for a controlled and reproducible reduction in cerebral blood flow, which is essential for the chronic cerebral hypoperfusion model.

BBR administration was based on previous studies, with the Sham + BBR and BCAS + BBR groups receiving BBR (Sigma, USA) at 30 mg/kg/day via intraperitoneal injection for six weeks starting immediately after surgery. The dosage of 30 mg/kg/day was selected based on its proven effectiveness in previous animal studies assessing cognitive deficits. The Sham and BCAS groups received an equivalent volume of normal saline daily.

Intracerebroventricular administration of AG490 (Merck, Germany) and Colivelin (Abmole, USA) was performed after anesthetizing the rats with pentobarbital sodium. The rats were placed on a stereotactic device to locate the bregma. The injection site was positioned 0.8 mm posterior and 1.5 mm lateral to the right of the sagittal suture, with a needle depth of 4.8 mm. A microinjector was used to administer 2 µL of a 10 mmol/L solution of AG490 or Colivelin. All injections were performed once every two days for six weeks, based on prior studies that reported significant effects on JAK2-STAT3 signaling with this regimen.

All experiments commenced six weeks after surgery. In addition to these groups, 30 rats were initially included as reserve animals to account for potential attrition or exclusion due to factors such as surgical complications, post-operative mortality, or abnormal behavior. However, no rats were excluded from the final analysis, and these reserve animals were not needed for replacement. Therefore, the final number of rats used in the analysis was 90, with 15 rats in each of the six experimental groups.

### 2.3. Morris water maze test

Ten rats were randomly assigned to each group and tested six weeks after the intervention using the Morris water maze to determine how well the rats learned and remembered. The experimental training phase lasted three days and involved training the rats four times a day. The rats were placed in one of four prearranged quadrants, with their backs to the pool wall. The time it took the rat to find and stay on a concealed underwater platform was recorded. After 60 s, if the rat still had not found the platform, it was gently taken there and given 10 s to relax. In the probe trial that followed training, escape latency—the amount of time for rats to find and climb the platform—was measured by introducing them to the pool from four fixed entry locations and timing how long it took them to do so within 2 min. This was maintained for a duration of six days, after which the average duration was determined. To evaluate the rats' spatial memory, spatial probe tests were used, which involved taking the platform away and counting how many times the rats ran by its old spot in 180 s. Rats were wiped dry with a towel after each session, and environmental factors like light and stillness were preserved.

### 2.4. NOR test

Subjects were placed in a controlled environment for a 5-min object recognition task. An overhead observational device recorded the behaviors in the vicinity. An interaction with an object was defined as the animal's nose being oriented toward or making contact with the object within approximately 2 cm. The exploration durations for object B (novel) and object C (familiar) were recorded. Cognitive function was assessed using the Preference Index, calculated using the following formula:

$$\text{Preference Index}=\left(\frac{\text{Time spent exploring novel objects}-\text{Time spent exploring familiar objects}}{\text{Total exploration time}}\right)\times 100\text{\%.}$$ (1)

### 2.5. Nissl staining

Five rats from each group were subjected to a 4% paraformaldehyde perfusion. After 48 h, brain tissues were fixed in the same solution. The brain tissue blocks were subsequently embedded using conventional methods and sectioned into 5 µm thick slices on a microtome. To facilitate attachment, slides were dried and placed in an oven at 45 °C. Following dewaxing in xylene, sections were rehydrated through graded ethanol solutions (100%, 95%, 90%, 70%, 50%). The slides were thereafter stained with 1% toluidine blue at 60 °C for 40 min. After staining, slides were cleaned in xylene and mounted with a coverslip. The sections were then dehydrated through a series of ethanol washes, with gradually increasing ethanol concentrations. Neuronal density in the CA1 region of the hippocampus was quantified using ImageJ software.

### 2.6. TUNEL staining

Before being stained with Nissl, sections were dewaxed, hydrated, and then washed three times with PBS. After that, they were treated with 20 µg/mL of DNase-free protease K (20 mg/mL) in P0106 immunostaining wash solution at 37 °C for 30 min. Sections were left to incubate in the dark at 37 °C for 60 min after three more PBS washes, after which 50 µL of TUNEL test solution was added. Before being studied under a fluorescence microscope, slices were sealed with anti-fading mounting solution and subjected to three more PBS washes. The number of cells that tested positive for TUNEL was recorded.

### 2.7. Western blotting (WB)

Brain tissue samples were taken from five rats per group. Samples were treated with 200 µL of detergent lysis solution (RIPA buffer with protease inhibitors) for 30 min on ice. The supernatant was collected after centrifugation at 12,000 rpm for 20 min at 4 °C. Protein concentrations were determined using a BCA protein assay (Pierce, Thermo Fisher Scientific), and the sample protein was adjusted to equal concentrations for electrophoresis. Gel percentage for SDS-PAGE was 12%. Boiling the protein solution with loading buffer for 5 min was the first step in preparing the sample. 5 µL of protein marker was loaded onto prepared gels, and SDS-PAGE was run at 80 V for 2.5 h. After electrophoresis, proteins were transferred to PVDF membranes using a wet transfer method at 18 V for 18 min. Membranes were soaked in 5% skim milk solution for 2 h to block nonspecific binding, followed by incubation with primary antibodies (cleaved caspase-3, JAK2, p-JAK2, STAT3, p-STAT3) overnight at 4 °C. After washing, secondary antibodies coupled with horseradish peroxidase were applied for 2 h. Protein bands were developed using an ECL kit (Pierce, Thermo Fisher Scientific), and images were captured using a gel documentation system. The ImageJ software was used to quantify the intensity of protein bands.

### 2.8. Molecular docking

The 3D structure of BBR was obtained and subjected to energy minimization using ChemBio3D Ultra 14.0 software, and the optimized structure was saved in mol2 format. The STAT3 protein structure was processed using PyMOL 2.3.0 and prepared for docking using AutoDock Tools (ver. 1.5.6).

### 2.9. Surface plasmon resonance (SPR)

To evaluate the binding affinity between recombinant STAT3 protein and BBR, a surface plasmon resonance (SPR) assay was conducted using a BIAcore T200 instrument (BIAcore, Cytiva). Binding affinity was quantified by calculating the dissociation constant (KD) values using the BIAcore T200 analysis software.

### 2.10. Statistical analysis

Statistical analysis was conducted using GraphPad Prism 8.0 and SPSS 23.0 software. Data were expressed as mean ± standard error of the mean (SEM). To evaluate differences among multiple experimental groups, one-way analysis of variance (ANOVA) was performed, followed by Tukey's post-hoc test for pairwise comparisons between all groups. For experiments involving only two groups, an independent samples t-test was used. A significance level of *P* < 0.05 was considered statistically significant. Specific significance markers are as follows:

*P < 0.05, **P < 0.01, ***P < 0.001 for comparisons against the Sham group;
- ^#^*P* < 0.05, ^##^*P* < 0.01, ^###^*P* < 0.001 for comparisons against the BCAS group.

For each behavioral and histological analysis, data were collected from 15 animals per group. In the western blot analysis, 5 animals per group were used to generate the tissue samples. Behavioral tests, histological analysis, and western blotting were performed using different sets of animals to avoid any potential interference between the assays, ensuring that the same animal was not used for multiple experiments.

## 3. Results

### 3.1. BBR improves cognitive impairment in BCAS rats

**Figure 1.**
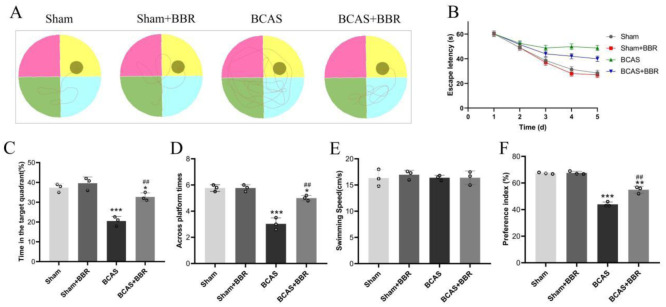
Effects of BBR on cognitive impairment in BCAS rats. A: Swimming trajectories of rats in the water maze. B: Escape latency during the water maze test. C: Percentage of time spent in the target quadrant. D: Frequency of platform crossings in the target quadrant. E: Swimming speeds of rats across all experimental groups. F: Preference Index from the NOR test.

Cognitive function in BCAS rats was assessed by measuring cognitive impairment and evaluating the efficacy of BBR treatment. Spatial learning and memory were assessed using the water maze, where BCAS rats exhibited prolonged escape latency. However, BBR treatment significantly reduced this latency (Figure 1A,B). In addition, during spatial exploration tasks, BCAS rats spent less time swimming and exhibited fewer platform crossings within the target quadrant compared to BBR-treated rats, who demonstrated increased swimming duration and platform crossing frequency in the target area (Figure 1B–D). No significant differences were observed between the Sham and Sham + BBR groups in these measures, nor in swimming speeds across all experimental groups. The NOR test further assessed cognitive performance, revealing a significantly lower Preference Index in BCAS rats compared to Sham rats, with BBR treatment leading to a notable improvement in this index (Figure 1F).

### 3.2. BBR inhibits neuronal damage and apoptosis in the hippocampus of BCAS rats

**Figure 2.**
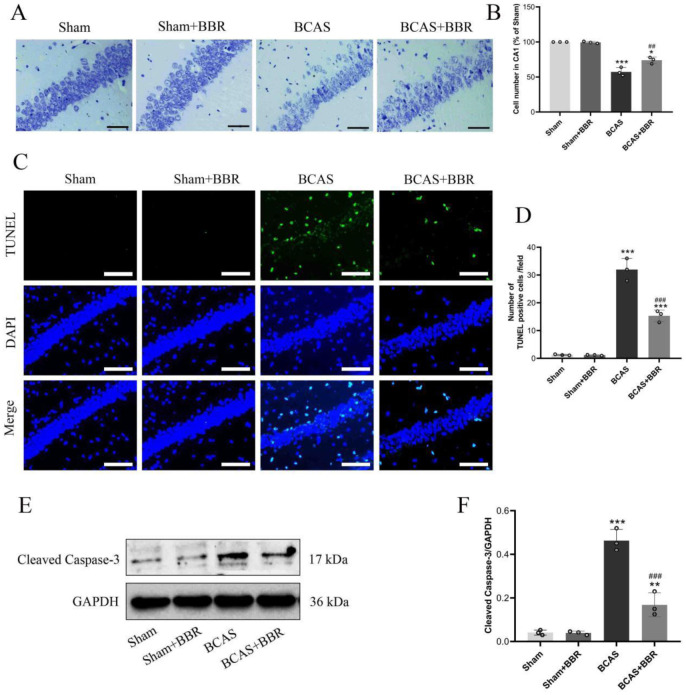
Protective effect of BBR on hippocampal neuronal damage in BCAS rats. A: Nissl staining of hippocampal CA1 region neurons. B: Statistical analysis of neuronal numbers in the CA1 region. C: TUNEL staining to assess apoptosis in the hippocampus. D: Expression of cleaved caspase-3, detected by western blot (WB). E: Semi-quantitative analysis of cleaved caspase-3 expression.

Nissl staining indicated a significant neuronal reduction and elevated apoptosis levels in the hippocampal CA1 region of BCAS rats. However, BBR treatment markedly increased the number of hippocampal neurons and reduced apoptosis in this region (Figure 2A–E). These results demonstrated that BBR could enhance the survival of hippocampal neurons in BCAS rats and exert neuroprotective effects.

### 3.3. BBR reduces hippocampal microglial activation and neuroinflammation in BCAS rats

**Figure 3.**
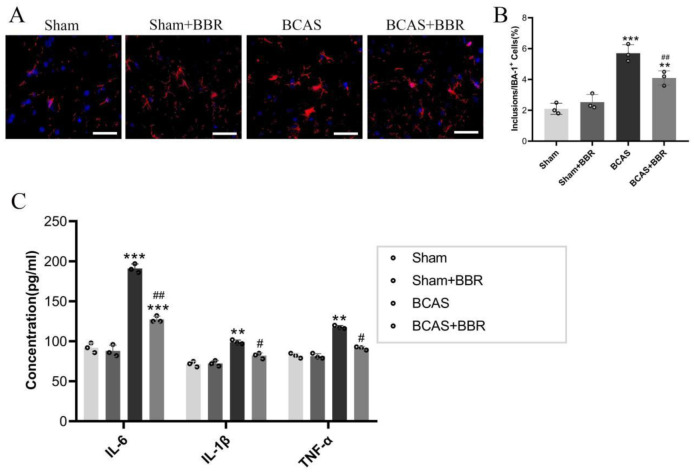
Effect of BBR on hippocampal neuroinflammation in BCAS rats. A: IBA-1 immunofluorescence staining of hippocampal microglia. B: Count of IBA-1 positive cells in the hippocampus. C: Expression levels of pro-inflammatory cytokines IL-6, IL-1β, and TNF-α in the hippocampus.

Morphological examinations of hippocampal microglia through immunofluorescence staining showed increased activation and release of inflammatory factors (IL-6, IL-1β, and TNF-α) in BCAS rats compared to Sham rats. BBR treatment significantly reduced microglial activation and the release of these inflammatory factors (Figure 3A–C).

### 3.4. Prediction of BBR interaction targets

**Figure 4.**
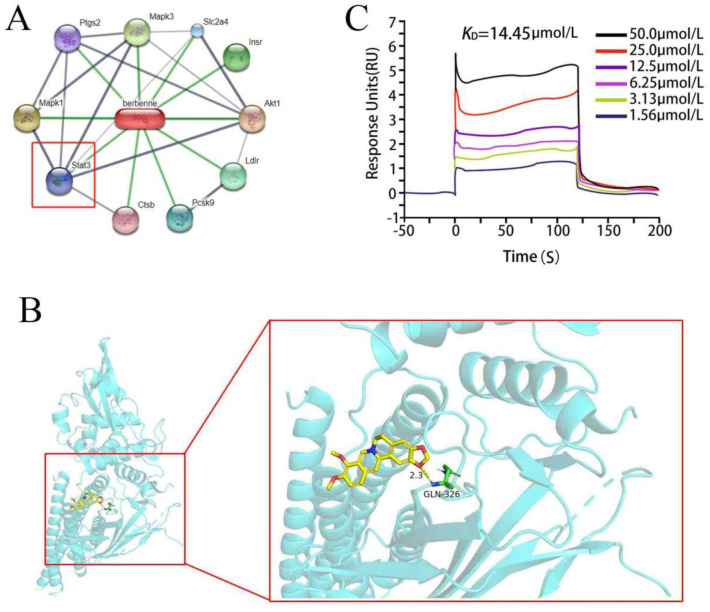
Prediction and binding of BBR to STAT3. A: Interaction prediction of BBR with potential targets from the STITCH database. B: Molecular docking of BBR with the STAT3 protein. C: Surface plasmon resonance (SPR) assessment of BBR binding affinity to STAT3.

To investigate the mechanisms underlying BBR's beneficial effects, we utilized the STITCH database to identify potential BBR targets and predicted the benefits of targeting STAT3 based on interaction scores (Figure 4A). Molecular docking experiments showed that BBR's binding affinity to STAT3 was −7.592 kcal/mol, indicating strong binding activity (Figure 4B). BBR interacts with STAT3 primarily through hydrogen bonds with THR-86 and GLN-65, measuring 2.3 and 2.6 Å, respectively, and hydrophobic interactions with VAL-62, ASN-78, TYR-102, ASP-102, LEU-93, and TRP-40. Additionally, in vitro SPR assays confirmed direct binding between BBR and STAT3, with an average KD value of 8.364E-5, demonstrating high binding affinity (Figure 4C).

### 3.5. JAK2/STAT3 pathway's role in BBR's effects on cognitive impairment in BCAS rats

**Figure 5.**
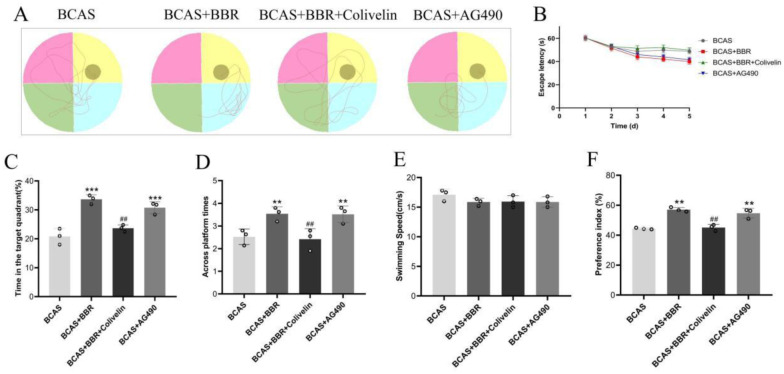
STAT3's role in BBR's modulation of cognitive impairment in BCAS rats. A: Swimming trajectories of rats in the water maze. B: Escape latency during the water maze test. C: Percentage of time spent in the target quadrant. D: Frequency of platform crossings in the target quadrant. E: Swimming speeds of rats across all experimental groups. F: Preference Index from the NOR test.

To confirm JAK2/STAT3 involvement in BBR's protective effects against cognitive impairment in BCAS rats, we administered the JAK2 inhibitor AG490 and the STAT3 activator Colivelin along with BBR. Cognitive abilities were evaluated using the water maze and NOR tests. The results indicated that both AG490 and BBR reduced escape latency and increased swimming time and platform crossing frequency in the target quadrant (Figure 5A–D). Interestingly, Colivelin reversed BBR's protective effects. In the NOR test, both AG490 and BBR increased the Preference Index of BCAS rats, while Colivelin again reversed BBR's protective effects (Figure 5F).

### 3.6. STAT3 contributes to BBR's protective effects on hippocampal neuronal damage in BCAS rats

**Figure 6.**
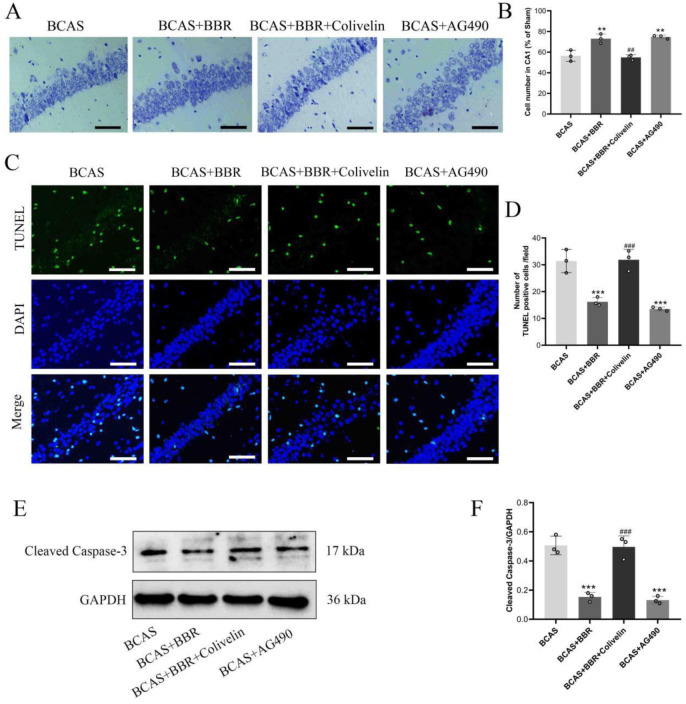
Involvement of STAT3 in BBR's impact on hippocampal neuronal damage in BCAS rats. A: Nissl staining of hippocampal CA1 region neurons. B: Statistical analysis of neuronal numbers in the CA1 region. C: TUNEL staining for apoptosis assessment in hippocampal neurons. D: Expression of cleaved caspase-3 detected by western blot (WB). E: Semi-quantitative analysis of cleaved caspase-3 expression.

For hippocampal neurons in the CA1 sector, treatments with BBR and AG490 not only augmented neuronal count but also curtailed apoptosis significantly (Figure 6A–E). On the contrary, Colivelin administration negated the neuroprotective effects of BBR.

### 3.7. STAT3's role in BBR's inhibition of hippocampal neuroinflammation in BCAS rats

**Figure 7.**
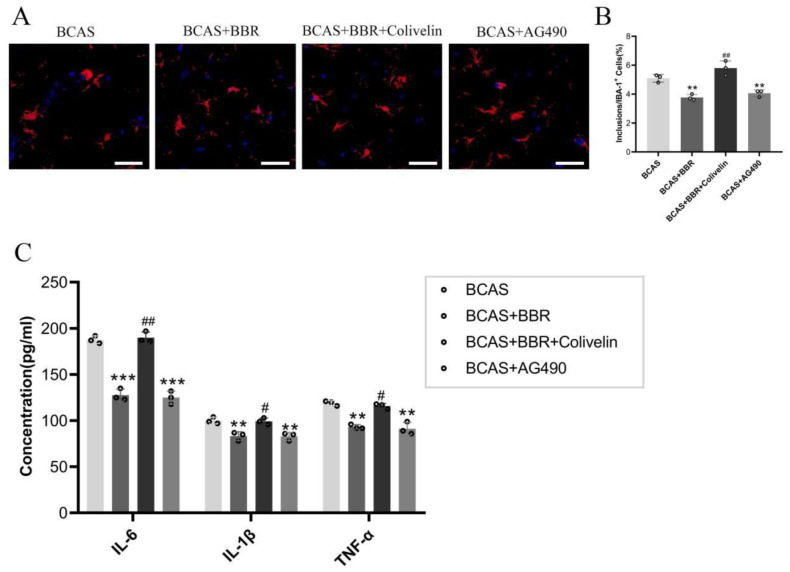
STAT3's role in BBR's inhibition of hippocampal neuroinflammation in BCAS rats. A: IBA-1 immunofluorescence staining of hippocampal microglia. B: Count of IBA-1 positive cells in the hippocampus. C: Expression levels of IL-6, IL-1β, and TNF-α in hippocampal tissue.

Treatments with BBR and AG490 notably reduced the activation of hippocampal microglia and the secretion of pro-inflammatory cytokines (IL-6, IL-1β, and TNF-α) (Figure 7A–C). Conversely, the inhibitory actions of BBR were countered by the administration of Colivelin.

### 3.8. Activation of the JAK2/STAT3 pathway counteracts BBR's neuroprotective effects

**Figure 8.**
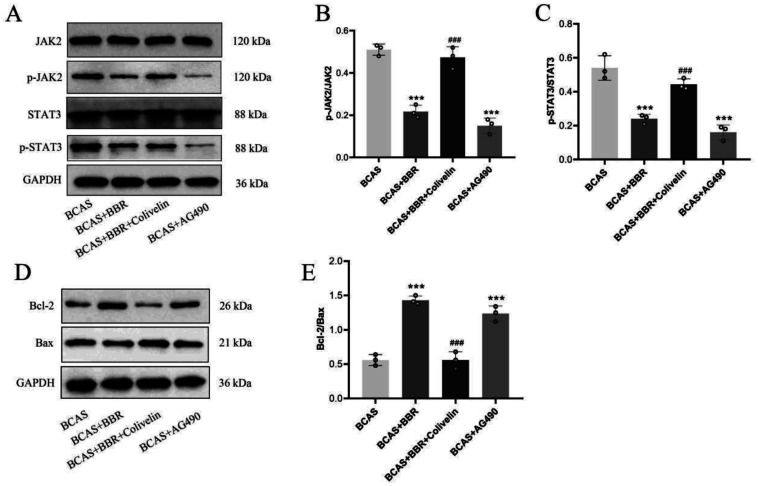
Impact of JAK2/STAT3 pathway activation on BBR's neuroprotective effects. A: Western blot (WB) analysis of phosphorylated JAK2 and STAT3 (p-JAK2, p-STAT3) in hippocampal tissue. B: Expression of p-JAK2 protein. C: Expression of p-STAT3 protein.

Similarly, these treatments led to a substantial decrease in the levels of phosphorylated JAK2 and phosphorylated STAT3 proteins in the hippocampus (Figure 8A–C). However, the introduction of Colivelin negated the neuroprotective impacts of BBR.

## 4. Discussion

Hippocampal formation, situated within the temporal lobe, is integral to the limbic system and is crucial for advanced cognitive functions, including learning and memory. Susceptible to hypoxic conditions, the hippocampus can experience atrophy, neuronal degeneration, and apoptosis under chronic cerebral ischemic conditions [20],[21]. The Morris water maze, introduced by the British psychologist Morris in the early 1980s, is an established method for evaluating animal learning and memory. It measures the time and path rats take to find a submerged platform in opaque water, being widely used in studies on learning, memory, AD, aging, and cognitive functions [22]. In this study, BCAS rats exhibited significantly longer escape latencies and spent less time in the target quadrant, indicating compromised spatial memory and learning abilities and confirming the effectiveness of the model.

BBR, a traditional anti-inflammatory compound, exhibits a range of biological effects, including antibacterial, anti-tumor, and anti-apoptotic activities and strong neuroprotection. It has been effectively used to treat cognitive impairments caused by various diseases [23],[24]. Six weeks after surgery, initial results from the positioning navigation test indicated no significant differences in escape latencies among the sham, BCAS, and BBR-treated groups on the first day. Subsequent tests, however, revealed that while the sham group quickly learned the platform's location, the BCAS group continued to show prolonged latencies. Spatial exploration tests further demonstrated that BCAS rats spent considerably less time in the quadrant previously containing the platform, highlighting deficits in spatial memory and navigation. Post six weeks of BBR administration, a marked improvement was noted in both escape latency and time spent in the target quadrant among the BBR group, signifying substantial cognitive enhancements. Nissl and TUNEL staining of hippocampal neurons confirmed that BBR treatment effectively mitigated neuronal damage and apoptosis induced by BCAS.

The JAK2/STAT3 signaling axis is a crucial intracellular pathway involved in various cellular functions. However, its precise role in CCH and cognitive deficits remains a subject of ongoing research. Ordinarily, activation of this pathway is transient, typically persisting for a duration ranging from several minutes to hours before undergoing rapid deactivation. However, in diseased states, this regulatory balance is disrupted, leading to sustained and atypical activation of the JAK2/STAT3 pathway. Such dysregulation is commonly linked to the development, progression, and prognosis of various pathological conditions, including inflammation, neoplastic diseases, and autoimmune disorders [25]–[28]. The JAK2/STAT3 signaling pathway exerts its effects through the activation of several downstream molecules that are key in mediating cellular survival, inflammation, and apoptosis. Upon phosphorylation, STAT3 translocates to the nucleus where it acts as a transcription factor, regulating the expression of various genes involved in cell survival and immune responses. Among the key downstream targets of STAT3 are genes encoding anti-apoptotic proteins such as Bcl-2, Bcl-xL, and Mcl-1, which play a crucial role in inhibiting mitochondrial-dependent apoptosis. Additionally, STAT3 activation leads to the upregulation of cyclin D1 and Cdk4, promoting cell cycle progression and neuronal survival. The anti-inflammatory effects of STAT3 are mediated by the suppression of pro-inflammatory cytokines such as IL-6, TNF-α, and IL-1β, which are known to contribute to neuroinflammation in conditions like BCAS. Moreover, STAT3's role in modulating microglial activation and its impact on neuroinflammation further support its central role in neuroprotection. The JAK2/STAT3 pathway's involvement in mitigating neuroinflammation is particularly important as it reduces the activation of microglial cells, which, when chronically activated, can exacerbate neuronal damage. In addition, the inhibition of this pathway by AG490 and the reversal of BBR's effects by Colivelin (a STAT3 activator) underscore the direct link between JAK2/STAT3 activation and the observed anti-inflammatory and neuroprotective effects in BCAS rats. The combination of anti-apoptotic and anti-inflammatory actions likely accounts for the significant cognitive improvements seen with BBR treatment. These findings highlight the potential of targeting JAK2/STAT3 signaling as a therapeutic strategy for neurodegenerative diseases and cognitive disorders associated with chronic cerebral ischemia.

Previous research has demonstrated that JAK2 and STAT3 are involved in synaptic plasticity and cognitive functions, while their direct impact in the context of BCAS-induced cognitive decline requires further validation [29]. As such, the implication of this pathway in disorders of the central nervous system (CNS), such as AD, has garnered increasing scholarly focus. In this experimental analysis, data showed that activation levels of p-JAK2 and p-STAT3 proteins were significantly elevated in the rat hippocampus under BCAS conditions compared to controls, which correlated with an increase in neuronal apoptosis. The use of AG490, a targeted JAK2 inhibitor, effectively reduced the phosphorylation of JAK2 and subsequent STAT3 activation, disrupting the signal transduction process. Administration of AG490 into the lateral ventricle in BCAS-afflicted rats resulted in decreased levels of p-JAK2 and p-STAT3 and a significant reduction in neuronal apoptosis. Additionally, Morris water maze assessments indicated enhancements in memory and learning capabilities in these treated animals. Therefore, the JAK2-STAT3 pathway is essential in mediating cognitive deficits triggered by BCAS.

Investigation of the effects of BBR in BCAS rats revealed that BBR treatment reduced the phosphorylation of JAK2 and STAT3 proteins, highlighting a potential neuroprotective effect. However, the direct interaction between BBR and STAT3 remains speculative, as the docking and SPR data did not conclusively confirm this interaction. Nevertheless, these effects were mitigated by the introduction of the STAT3 activator Colivelin into the lateral ventricle, confirming that the protective effects of BBR are mediated via the JAK2-STAT3 pathway. One limitation of this study is the lack of direct analysis of key downstream target genes of the JAK2/STAT3 pathway, particularly those involved in apoptosis (e.g., Bcl-2, Bax) and inflammation. While the results demonstrated that JAK2/STAT3 signaling could play a central role in mediating neuroprotective effects, the expression levels of these downstream effectors were not directly assessed. Future studies will benefit from quantifying the expression levels of these specific genes to further validate the mechanistic pathways underlying the anti-apoptotic and anti-inflammatory effects.

While the present findings in BCAS rats provide promising insights into the neuroprotective effects of BBR, translating these results into clinical practice for treating cognitive disorders presents both opportunities and challenges. One of the key strengths of BBR as a potential therapeutic agent is its well-established safety profile in humans, given its long history of use in traditional medicine. Moreover, the compound's multi-target actions, particularly its effects on neuroinflammation and apoptosis, are promising for conditions such as AD and other forms of cognitive decline. However, there are several challenges that must be addressed before BBR can be effectively translated into clinical treatments. First, the JAK2/STAT3 signaling pathway, though a promising target, is involved in a wide range of physiological processes, and its modulation may have unintended effects in non-CNS tissues. Therefore, further research is needed to delineate the specific role of JAK2/STAT3 signaling in different cellular contexts, particularly in humans, to minimize any potential off-target effects. Additionally, while BBR has shown efficacy in animal models, the appropriate dosing regimen and pharmacokinetic properties of BBR need to be thoroughly evaluated in clinical trials. It is also crucial to assess the long-term safety of BBR in humans, especially for chronic use in older populations, who are more likely to suffer from cognitive impairment. Finally, while BBR has shown robust effects in rodent models, the complexity of human cognitive disorders, which often involve multiple genetic and environmental factors, necessitates a more detailed approach. The potential for personalized medicine approaches, such as selecting patients based on specific biomarkers of JAK2/STAT3 activation, may enhance the clinical efficacy of BBR.

In conclusion, BCAS leads to cognitive decline in rats by inducing activation of the JAK2-STAT3 pathway and apoptosis of hippocampal neurons. While BBR appears to mitigate neuronal apoptosis and cognitive deficits, further studies are needed to conclusively confirm its mechanism of action, particularly in relation to the JAK2-STAT3 pathway and its downstream signaling components, such as Bcl-2, Bax, and MAPK pathways. Thus, targeting the BBR and JAK2-STAT3 pathway offers a new avenue for treating cognitive impairment resulting from BCAS, although the specific mechanisms of its downstream pathways require further exploration and validation.

## 5. Conclusions

This research suggests that BBR enhances cognitive functions in BCAS rats predominantly by reducing apoptosis in hippocampal neurons through the modulation of the JAK2/STAT3 pathway.

## Use of AI tools declaration

The authors declare they have not used Artificial Intelligence (AI) tools in the creation of this article.
